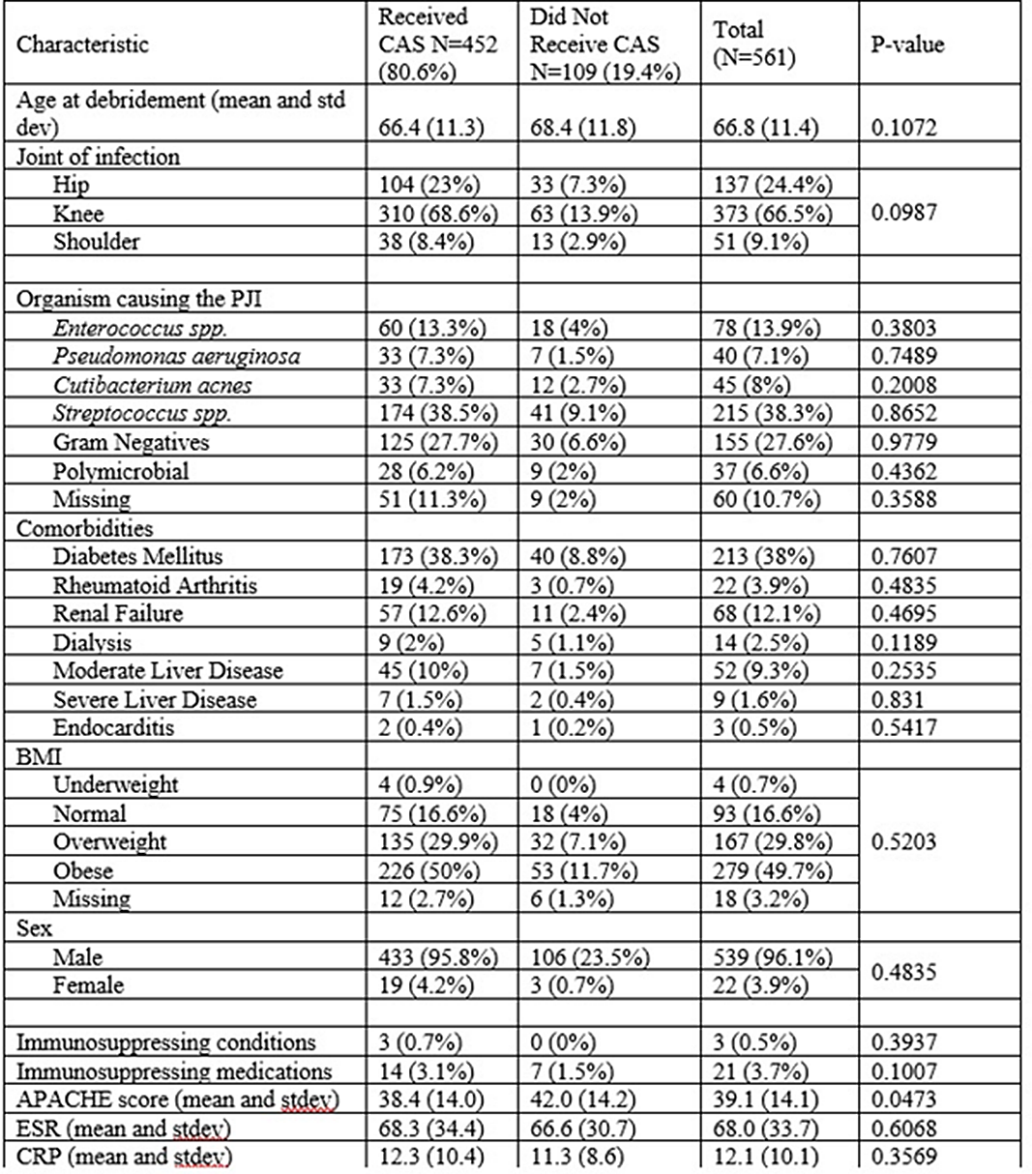# Chronic Antibiotic Suppression for Nonstaphylococcal Prosthetic Joint Infections Treated with Debridement or Implant Retention

**DOI:** 10.1017/ash.2021.8

**Published:** 2021-07-29

**Authors:** Poorani Sekar, Rajeshwari Nair, Brice Beck, Bruce Alexander, Kelly Miell, Aaron Tande, Kimberly Dukes, Julia Friberg, Marin Schweizer, Andrew Pugely

## Abstract

**Background:** Early postoperative and acute prosthetic joint infection (PJI) may be managed with debridement, antibiotics, and implant retention (DAIR). Among patients with nonstaphylococcal PJI, an initial 4–6-week course of intravenous or highly bioavailable oral antibiotics is recommended in the Infectious Diseases Society of America (IDSA) guidelines, with disagreement among committee members on the need for subsequent chronic oral antimicrobial suppression (CAS). We aimed to characterize patients with nonstaphylococcal PJI who received CAS and to compare them to those who did not receive CAS. **Methods:** This retrospective cohort study included patients admitted to Veterans’ Affairs (VA) hospitals from 2003 to 2017 who had a PJI caused by nonstaphylococcal bacteria, underwent DAIR, and received 4–6 weeks of antimicrobial treatment. PJI was defined by Musculoskeletal Infection Society (MSIS) 2011 criteria. CAS was defined as at least 6 months of oral antibiotics following initial treatment of the PJI. Patients were followed for 5 years after debridement. We used χ^2^ tests and *t* tests were used to compare patients who received CAS with those who did not receive CAS. **Results:** Overall, 561 patients had a nonstaphylococcal PJI treated with DAIR, and 80.6% of patients received CAS. The most common organisms causing PJI were streptococci. We detected no significant differences between patients who received CAS and those who did not receive CAS, except that modified Acute Physiology and Chronic Health Evaluation (mAPACHE) scores were higher among patients who did not receive CAS (Table 1). **Conclusion:** Patients not on CAS were more severely ill (by mAPACHE) than those on CAS. Otherwise, the 2 groups were not different. This finding was contrary to our hypothesis that patients with multiple comorbidities or higher mAPACHE scores would be more likely to get CAS. A future analysis will be conducted to assess treatment failure in both groups. We hope to find a specific cohort who may benefit from CAS and hope to deimplement CAS in others who may not benefit from it.

**Funding:** No

**Disclosures:** None

**Table 1.**